# Supplementary material for: A Portrait of the Rights of Children with Disabilities in Nigeria: A Policy Review
Source: Int J Environ Res Public Health. 2023 Oct 30;20(21):6996. doi: 10.3390/ijerph20216996 (PMC10650762; doi:10.3390/ijerph20216996)
Supplement: Supplementary file 1 [file ijerph-20-06996-s001.zip › ijerph-2574194-supplementary.pdf]

A portrait of the rights and disability rights of children in Nigeria: A policy review

Table S1. Nigerian List of Obstacles to the CRC.

Reports / Policy Documents	Details of Obstacles
The Nigerian CRC Summary Record 2010	- The Government did not allocate specific budget resources to children but the Ministry of Health or the Ministry of Education, is to provide a budget line for children's issues.<br>- Although progress had been made, children continue to be hindered in the exercise of their rights by Nigerians' belief that education should be authoritarian. It was therefore still difficult to set up children's rights clubs in schools.<br>- A study carried out in 2009 revealed inadequacies in the education and health of persons with disabilities, which had led the Government to formulate national action plans to address the issue with another study to assess the current situation of children with disabilities and was set to be completed by the end of 2010.
The Nigerian CRC Concluding Observations 2010	- The Committee regrets that a comprehensive review on the compatibility of the existing statutory, with the Convention and the Child Rights Act has not been carried out wishing to reiterate its earlier concern about insufficient budget allocations to children with corruption still an endemic in the State party and its adverse effects on the protection and promotion of children's rights.<br>- The concerns about the limited participation of children in matters affecting them in children's institutions of all kinds, in the community, in the family and in judicial and administrative procedures.<br>- There are also regrets about the low level of acceptance thereof, especially in rural areas, and appreciates the State party's acknowledgement of this situation.<br>- The proposed framework in the National Economic Empowerment and Development Strategy (NEEDS II) was welcomed for providing support to vulnerable groups (single parents and teenage mothers), there are regrets on the lack of information on measures taken to support parents, guardians or members of the extended family.<br>- There is also no data concerning single-parent families and is concerned about the reported social stigmatization facing single mothers.<br>- The slow process of adoption of the Child Rights Act 2003.<br>- Apparent lack of coordination, sufficient resources allocated to, and authority vested in the Department of Child Development, Federal/State Ministries of Women Affairs/NCRIC and NHRC entrusted with monitoring compliance with the Convention, lack of information about a comprehensive, rights-based, and time-framed National Plan of Action.<br>- Inadequate information about measures to address the severe lack of financial resources allocated to the protection and promotion of children's rights/maximum extent of available resources, lack of information on a systematic plan to introduce training and awareness among professional groups working for and with children.
The Nigerian CRC Reply to LOI 2010	- Childcare givers and child justice administrators in most states that have passed the child's rights laws are ill-equipped and lack the capacities to provide recovery services for the thousands of vulnerable children living in the streets and in institutions including those in conflict with the law and those in need of special care and attention.<br>- Also, there is weak institutional capacity for effective implementation and monitoring of child rights issues and programmes. Therefore, it is difficult to isolate such allocations, or fully account for all expenditures in respect to the proportion of the

	budget devoted to social expenditures for children in the areas of health, welfare, social services, recreation, and leisure.<br>- Insufficient budgetary allocation in this area remains a challenge and government leadership are essential to ensure that national responses are properly resourced, coordinated, implemented, and monitored

Table S2. Nigerian List of Enablers to the Convention on the Rights of a Child.

Reports / Policy Documents	List of Enablers
Nigeran Child Survival Policy (2002)	- Child Policy draft provides broad-based direction to various levels of government, donor agencies, NGOs and the private sector, and other concerned stakeholders in survival, development, and total well-being of the Nigerian child.
Nigerian Child Rights Act (2003)	- The following institutions to be known as approved children's institutions to be established by the federal government; (i) a Children Attendance Centre, (ii) a Children Centre, (iii) a Children Residential Centre, (iv) a Children Correctional Centre, (v) a Special Children Correction Centre, and (vi) such other institutions as the Minister may, from time to time
The Nigerian CRC State Party Report (2008)	- Efforts have been made at the Federal, State and Local government levels in Nigeria to translate the provisions of the Convention into reality and ensure its effective practical implementation.
Nigerian CRC Concluding Observation(2010)	- Effective functioning of the Children's Parliaments.<br>- Implementation of its mandate to deliberate and contribute to draft child-oriented bills to ensure that their composition is representative of all segments of society, including orphans, children with disabilities, refugee children, and other children with special needs.
Nigerian CRC Reply to LOI(2010)	- Baseline data on various issues affecting the rights of children and youths in Nigeria through a National Baseline Survey.<br>- Data generated through the baseline survey is being employed to formulate laws, policies, and programmes for the effective implementation of the Convention.<br>- The main goal of the National Plan of Action by 2010 is to ensure mechanisms for the protection, care, and support of orphans and vulnerable children are in place with the provision of basic services to facilitate a supportive environment.<br>- Creation of a guideline for Protection of Children in Formal Institutions was developed to help practitioners and service providers perform appropriate care giving services to vulnerable children and child trafficking survivors.<br>- Provision of adequate budgetary allocations for programmes for children with disabilities; Monitoring and evaluation of progress achieved in the care of children with disabilities.<br>- More effective intervention strategies in favor of children with disabilities and organizations, to enlighten the general public on the plight of children with disabilities.<br>- Access to scholarship as well as, free medical care, school bus, recreation facilities and book subsidies in some states for children with disabilities.<br>- Timely data collection on issues relating to children with disabilities for more effective intervention, and the mechanisms being developed for early detection of disabilities, the creation of more schools for children with disabilities and the capacity building through the creation of more specialized teachers and training colleges with emphasis on disability.

The Nigerian CRC Summary Record (2010)	- Plans to carry out a study of the structural gaps at the local level, to launch initiatives to develop the capacity of key personnel, and to promote community participation, which could contribute greatly to improving the situation of children in Nigeria.<br>- Incorporation of the provisions of the Convention into domestic law by enacting the Child Rights Act, adoption of the Act by 24 states was encouraging, but with regret that 12 states still had not adopted it and the State party was asked to do its best to address the underlying causes of that delay.<br>- Further information on policies related to children with special needs (children with disabilities), with a request on fuller data on children with disabilities in rural and urban areas, in relation to their schooling.

Table S3. Nigerian List of Needs met for the Convention on the Rights of a Child.

Reports / Policy Documents	Needs Met
Nigerian Child Survival Policy (2002)	Adoption and implementation of a number of major global initiatives affecting children, such as the Safe Motherhood Initiative and its follow-up Making Pregnancy Safer, Baby-Friendly Hospital Initiative (BFHI), and Integrated Management of Childhood Illness (IMCI). Others are RBM Initiative, Elimination of IDD, VAD Control, and NPI, the latter with a special emphasis on the eradication of poliomyelitis.
Nigerian CRC Concluding Observations (2010)	The adoption of policies and strategies aiming at strengthening the implementation of the Convention, including:<br>(i) The National Plan of Action on CRC/CRA (2009-2015) of 2008;<br>(ii) The National Action Plan for the Promotion and Protection of Human Rights in Nigeria (2009-2013)<br>(iii) The National Child Policy and National Child Health Policy of 2007;<br>(iv) The National Plan of Action on Orphans and Vulnerable Children (20062010) of 2007.<br>(v) The National Policy and Guidelines for the Monitoring of Child Care Centers in Nigeria.<br>(vi) The National Policy on Adolescent Health of 2006 and the Integrated Maternal, Newborn and Child Health (IMNCH) Strategy of 2007;<br>(vii) The Action Plan developed by the National Population Commission for a permanent and sustainable birth registration system.

Table S4. Human Rights Law.

Reports / Policy Documents	Human Rights Law
Lagos Disability Bill, 2010	- The bill also noted that no person living with disability shall be subjected to prejudices or harmful practices, including those based on sex, work, age, or tradition in any area of life.<br>- Persons living with disability have the right to sue any individual, corporate body or Government and its Agencies for damages in the event of the violation of their fundamental rights.

	- The government is also required to provide free legal aid without condition to persons living with disability through the Office of the Public Defender (OPD) when required.<br>- No person living with disability shall be subjected to: (a) medical or scientific experiment without his or her (b) consent; or torture, cruelty, or inhuman degrading treatment.<br>- No person shall employ, use, or involve a person living with disability for alms begging. Any person in contravention of this section shall on conviction be liable to a fine of N100,000.00 (One Hundred Thousand Naira) or a maximum 3 (three) months imprisonment or both.
The Nigerian Child Rights Act 2003	- Where a parent, guardian or person who has care and custody of a child, fails in the duty imposed, commits an offence and is liable- (a) on first conviction to be reprimanded and ordered to undertake community service; (b) on second conviction to a fine of two thousand Naira or imprisonment for a term not exceeding one month or to both such fine and imprisonment.

Table S5. Details of Fundamental Rights.

Policy Documents	Government Responsibility	Examples
Nigerian Child Rights Act 2003	A State Government shall provide accommodation for every child.	- A child in need within the State who appears to require accommodation where there is no person having parental responsibility for the child.<br>- A child who is ordinarily resident in another State, State Government may take over the provision of accommodation for the child within three months of being notified in writing that the child is being provided accommodation.<br>- A child within the State whose welfare the appropriate authority considers is likely to be seriously prejudiced if the State Government does not provide the child with accommodation.<br>- A child under this section by placing him with a family, a relative of the child, any other suitable person, on such terms as to payment by the State Government and otherwise as the State Government may determine.
The Lagos State Disability Bill 2010	Actualizing the enjoyment of all rights in this Law by persons living with disability.	- Mobility aids and appliances or assistive devices used by passengers living with disability shall not be subjected to charges.<br>- No discrimination of person living with disability by any person or institution<br>- Persons living with disability shall be given consideration of 5% reservation on accommodation provided by schools, Employers, Organizations, and Government

	- No child shall be concealed, abandoned, neglected, or segregated on the basis of his or her disability.<br>- Persons living with disability shall have freedom of expression and opinion, through any means of communication of their choice.

Table S6. Other identified actions in Policy Review.

No	List of other identified actions of the Nigerian Government
1	Increase of budget allocations to health and education;
2	Investment of funds saved from the State Party's debt relief program into children's programs, which included the establishment of the Children's Parliament in all 36 states and their active participation in international as well as national forums.
3	Adoption of the National Plan of Action on Orphans and Vulnerable Children and the National Guidelines and Standards of Practice on Orphans and Vulnerable Children based on a rights-based approach and guided by the principle of the best interest of the child;
4	Adoption of health-related policies; provision of free maternal and child health services in 18 northern states and additional plan to increase its allocations in the health sector
5	Implementation of free basic education program and increased budgetary allocations for the education section and infrastructures
6	Adoption of the Vocational Educational Initiative and the development of special vocational training programs to assist children from low socio-economic status and for children from other vulnerable groups

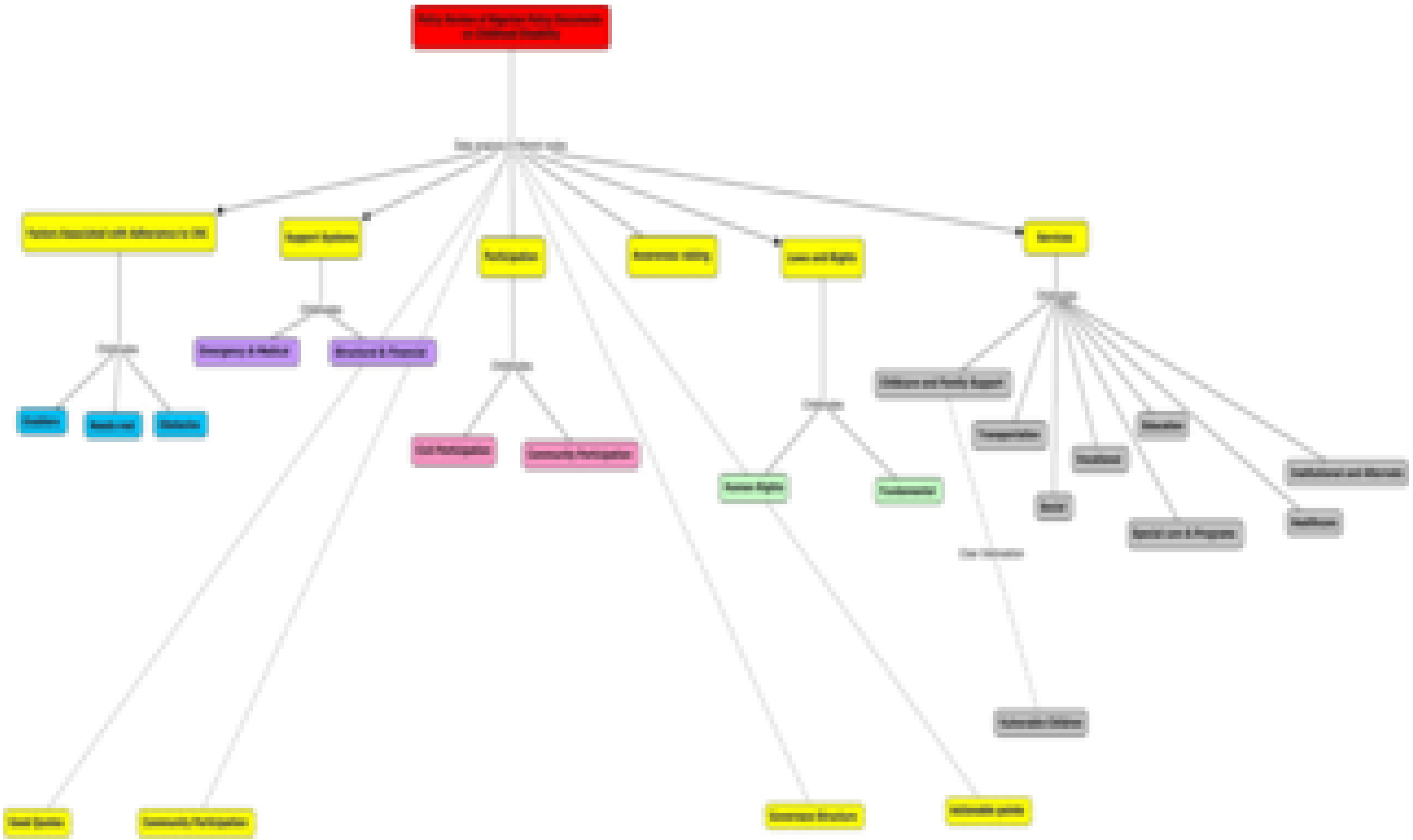

Figure S1. Themes and sub-themes from analysis.
